# Genome-Wide Identification and Gene Expression Analysis of ABA Receptor Family Genes in *Brassica juncea* var. *tumida*

**DOI:** 10.3390/genes10060470

**Published:** 2019-06-20

**Authors:** Chunhong Cheng, Yuanmei Zhong, Zhaoming Cai, Rongbin Su, Changman Li

**Affiliations:** School of Advanced Agriculture and Bioengineering, Yangtze Normal University, Chongqing 408100, China; xiaobei15109217512@163.com (C.C.); zgcqzym@126.com (Y.Z.); caizhaoming-2000@163.com (Z.C.); surongbincoffee@126.com (R.S.)

**Keywords:** *Brassica juncea* var. *tumida*, ABA receptor, *BjuPYLs*, genome-wide, gene expression

## Abstract

Abscisic acid (ABA) plays important roles in multiple physiological processes, such as plant response to stresses and plant development. The ABA receptors pyrabactin resistance (PYR)/ PYR1-like (PYL)/regulatory components of ABA receptor (RCAR) play a crucial role in ABA perception and signaling. However, little is known about the details regarding *PYL* family genes in *Brassica juncea* var. *tumida*. Here, 25 *PYL* family genes were identified in *B. juncea* var. *tumida* genome, including *BjuPYL3*, *BjuPYL4s*, *BjuPYL5s*, *BjuPYL6s*, *BjuPYL7s*, *BjuPYL8s*, *BjuPYL10s*, *BjuPYL11s*, and *BjuPYL13*. The results of phylogenic analysis and gene structure showed that the *PYL* family genes performed similar gene characteristics. By analyzing *cis*-elements in the promoters of those *BjuPYLs*, several hormone and stress related *cis*-elements were found. The results of gene expression analysis showed that the ABA receptor homologous genes were induced by abiotic and biotic stress. The tissue-specific gene expression patterns of *BjuPYLs* also suggested those genes might regulate the stem swelling during plant growth. These findings indicate that *BjuPYLs* are involved in plant response to stresses and organ development. This study provides valuable information for further functional investigations of *PYL* family genes in *B. juncea* var. *tumida*.

## 1. Introduction

The plant phytohormone abscisic acid (ABA) is involved in multiple plant physiological processes, such as seed dormancy, seed germination, seedling development, post-germinative growth, stomatal movement, and synthesis of storage proteins and lipids [1,2,3,4,5,6,7]. ABA also plays an essential role in plant responses to adverse environmental stresses, such as abiotic stresses (salinity and low-temperature stresses) and biotic stresses [1,2,8]. So far, the core components of the ABA signaling pathway have been identified in *Arabidopsis* [9,10,11]. The ABA signaling pathway mainly contains pyrabactin resistance (PYR)/PYR1-like (PYL) regulatory components of ABA receptor (RCAR) protein family (ABA receptors), the co-receptors group A protein type 2C phosphatases (PP2Cs), and sucrose nonfermenting-1-related protein kinase class 2 (SnRK2s) [9,10,11]. In the absence of ABA, PP2Cs interact with SnRK2s and inhibit their kinase activity, which resulted in turning off of ABA signaling pathway. In the presence of ABA, PYR/PYL/RCAR receptors bind the hormone ABA, allowing them to physically associate with PP2Cs and eliminate the inhibitory effect of the phosphatases on SnRK2s, which phosphorylate bZIP (basic-leucine zipper) transcription factors and ion channels to turn on the ABA signaling.

The ABA receptors PYR/PYL/RCAR are responsible for ABA perception and signaling [6,12]. In *Arabidopsis*, the ABA receptors contain 14 family members, which are PYR1, PYL1–PYL13. Recently, the mechanisms about how these ABA receptors regulate plant responses to ABA, abiotic stresses, and biotic stresses have been characterized. For example, overexpression of *NtPYL4* in tobacco hairy roots caused the reprogramming of the cellular metabolism, leading to a decreased alkaloid accumulation, and conferred ABA sensitivity to the production of alkaloids [13]. Overexpression of *RSL1* (RING FINGER OF SEED LONGEVITY1) in *Arabidopsis* resulted in decreased ABA sensitivity of plants by promoting the degradation of PYR1 and PYL4 [14]. OsPYL5, acting as an ABA receptor, can increase rice tolerance to salt and drought stresses [15]. In *Brassica napus*, the transcript levels of *BnPYLs* genes were analyzed by qPCR in plant roots under multiple abiotic stresses treatment, and the results showed that the gene expression levels of *BnPYR1-3*, *BnPYL1-2*, and *BnPYL7-2* were induced by drought, heat, and salinity treatments [16].

*B. juncea* var. *tumida* (AABB, 2n = 36), which belongs to Brassicaceae, is an allotetraploid species by a natural cross between *Brassica rapa* (AA, 2n = 20) and *Brassica nigra* (BB, 2n = 16) following with subsequent chromosome doubling [17]. *B. juncea* var. *tumida* is a kind of vegetable in China and some south-east Asia countries. The swollen stem (the diameter of the stem can swell to more than 20 cm) of *B. juncea* var. *tumida* is the raw material for Fuling mustard, which is famous for its special flavor and nutritional value [18]. However, little is known about the regulation mechanism of stem swelling. Tuber mustard always suffers from abiotic stresses (salinity and low-temperature) and biotic stress (*Plasmodiophora brassicae*), resulting in inhibition of plant growth and huge economic loss. The ABA receptors are key components in the ABA signaling pathway, mediating plant development and response to stresses. Identifying the ABA receptor family genes in *B. juncea* var. *tumida* is not only helpful for further understanding the ABA signaling pathway but also provides cues for the plant to defend against stresses. However, the role and function of the ABA receptor family genes in *B. juncea* var. *tumida* remains mainly unknown.

In this study, we identified 25 ABA receptor family genes in the *B. juncea* var. *tumida* genome. Based on the analysis of the phylogenic relationship, gene structures, and promoter *cis*-elements, similar gene characteristics were found between *BjuPYLs* and *AtPYLs*. The *BjuPYLs* expression patterns in different tissues were analyzed by qPCR, and the result showed that the 25 *BjuPYLs* have a different organ and tissue expression patterns. In addition, we analyzed the genes transcriptional levels of *BjuPYLs* under abiotic stresses (including NaCl, ABA, and low-temperature) and biotic stress (*P. brassicae*). We also analyzed the *BjuPYLs* transcriptional levels during different developmental stages of *B. juncea* var. *tumida* (the developmental stages of D1 to D5). The results showed that *BjuPYLs* were induced by abiotic and biotic stresses in *B. juncea* var. *tumida*, and *BjuPYLs* (*BjuPYL4-3*, *BjuPYL5-3*, *BjuPYL5-5*, *BjuPYL6-2*, *BjuPYL8-2*) might play roles in stem swelling.

## 2. Materials and Methods

### 2.1. Materials and Growth Conditions

Tuber mustard cultivar Yong’an was used for gene expression analysis. The seeds were surface sterilized and plated on MS medium (Sigma-Aldrich, St. Louis, MO, USA) with 1% sucrose and 8 g/L agar (Sigma-Aldrich, St. Louis, MO, USA) and then cultivated in growth room at 22 °C and 6000 lx under long-day conditions (16 h light/8 h dark) for seven days and then treated with 50 μM ABA, 200 mM NaCl, and at low-temperature (4 °C) for 3 h. For pathogen (*P. brassicae*) treatment, 2-week-old seedlings of *B. juncea* var. *tumida* were irrigated with *P. brassicae* suspension liquid (OD_600_ = 0.07) for the indicated time points.

### 2.2. Bioinformatics Analysis

The gene sequences of *AtPYLs* and their homologous genes in *B. juncea* var. *tumida* were searched in TAIR (http://www.arabidopsis.org/) and Brassica database (http://brassicadb.org/brad/). The phylogenic tree was constructed using the neighbor-joining method with the bootstrap values of 1000 by MEGA5 [19]. The gene structure analysis was done by online software (http://gsds.cbi.pku.edu.cn/). The protein sequences were aligned by ESPript 3.0 online software (http://espript.ibcp.fr/ESPript/cgi-bin/ESPript.cgi) and WebLogo online software (http://weblogo.berkeley.edu/). The promoter *cis*-element analysis was performed using online analysis software of PlantCARE (http://bioinformatics.psb.ugent.be/webtools/plantcare/html/) and PLACE (https://sogo.dna.affrc.go.jp/cgi-bin/sogo.cgi?lang=en&pj=640&action= page&page=newplace).

### 2.3. Gene Expression Analysis

Total RNA of different samples were extracted from *B. juncea* var. *tumida* seedlings using TRIzol reagent. The RNA samples were used for cDNA synthesis using a cDNA synthesis Supermix with gDNA remover kit (TransGen Biotech, Beijing, China) following the manufacturer’s instructions. qRT-PCR was carried out using SYBR Green qPCR Supermix (Invitrogen, Carlsbad, CA, USA). The transcript abundance was calculated by the comparative *C_T_* (cycle threshold) method, and *BjuActin3* was used as the internal control. The qRT-PCR experiments were carried out three times, each with three replicates. The primers used are listed in Table S1.

## 3. Results

### 3.1. Genome-Wide Identification and Characterization of BjuPYLs in *B. juncea* var. *tumida*

**Figure 1:**
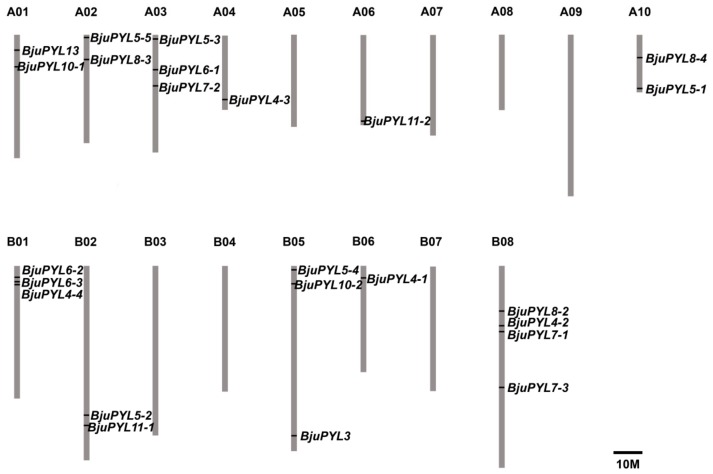
The distribution of *BjPYLs* in *B. juncea* var. *tumida* chromosomes. Twenty-five identified *BjuPYLs* were mapped to the 11 of 18 chromosomes. The chromosome name is at the top of each bar. The scale of the chromosome is in millions of bases (Mb).

25 BjuPYLs were identified in *B. juncea* var. *tumida* genome through BLASTP in *Brassica* database as homologs of AtPYLs by using nine *AtPYLs* protein sequences as references. No homologs were found for the proteins of AtPYR1, AtPYL1, AtPYL2, AtPYL9, and AtPYL12 (Table 1). The lengths of these ABA receptor genes ranged from 486 bp to 1238 bp with 1–3 exons in each sequence. The protein lengths of BjuPYLs ranged from 162 (BjuPYL11-1) to 221 (BjuPYL6-3) amino acid (aa) residues. The relative molecular weights of these proteins varied from 18.05 kD (BjuPYL11-1) to 24.25 kD (BjuPYL6-3), and the isoelectric point (PI) was 5.01–9.12 (Table 1). The 25 *BjuPYLs* genes were distributed in 11 of 18 chromosomes of *B. juncea* var. *tumida*. Each of the chromosomes A04, A06, B06 contained one gene; A01, A02, A10, B02 contained two genes; A03, B01, B05 contained three genes; the other four genes were all located in B08 (Figure 1).

### 3.2. The Phylogenic Analysis and Gene Structures of BjuPYLs

**Figure 2:**
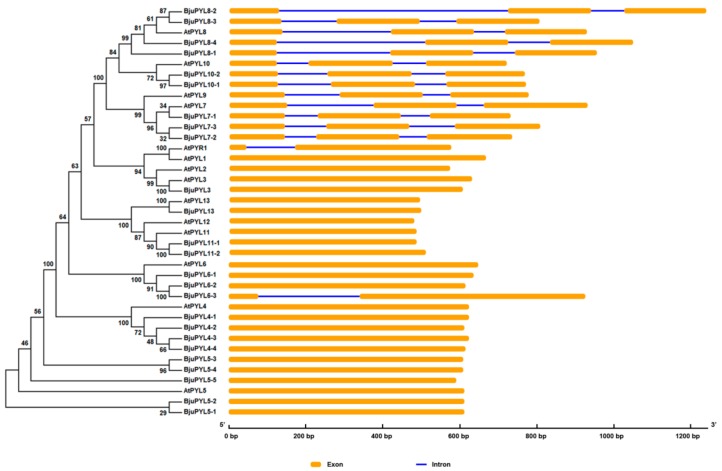
The phylogenic tree and gene structures of *BjuPYLs* and *AtPYLs*. The phylogenic tree was built with the neighbor-joining (NJ) method using the protein sequences, and the exon-intron structure of pyrabactin resistance (PYR)1-like (PYL) homologs was drawn according to their phylogenic relationships. The orange boxes and blue lines denote exons and introns, respectively.

To analyze the evolutionary relationships between *BjuPYLs* and *AtPYLs*, a phylogenetic tree was constructed by MEGA5 software with the neighbor-joining method using the protein and genomic sequences (Figure 2, Figure S1). According to the phylogenic tree, 25 *BjuPYLs* with 14 *AtPYLs* were identified. The *BjuPYL* genes were named following their homologs in *Arabidopsis* (Figure 2, Table 1, Figure S1). To understand the gene structures of *BjuPYLs*, the gene exon-introns were identified using the online software of GSDS2.0 server. According to the result, *BjuPYL3*, *BjuPYL4-1* to *BjuPYL4-4*, *BjuPYL5-1* to *BjuPYL5-5*, *BjuPYL6-1* to *BjuPYL6-2*, *BjuPYL11-1* to *BjuPYL11-2*, and *BjuPYL13* all had one exon; *BjuPYL6-3* had two exons; *BjuPYL7-1* to *BjuPYL7-3*, *BjuPYL8-1* to *BjuPYL8-4*, and *BjuPYL10-1* to *BjuPYL10-2* all contained three exons (Figure 2). Almost all the *AtPYLs* had the same gene structures with their homologs genes in *B. juncea* var. *tumida*, except *BjuPYL6-3*, which had two exons, while *AtPYL6* had one exon. These results indicated that the *BjuPYLs* shared similar gene structures with their homologs in *Arabidopsis*.

### 3.3. The Alignment of PYL Proteins and Motif Analysis

**Figure 3:**
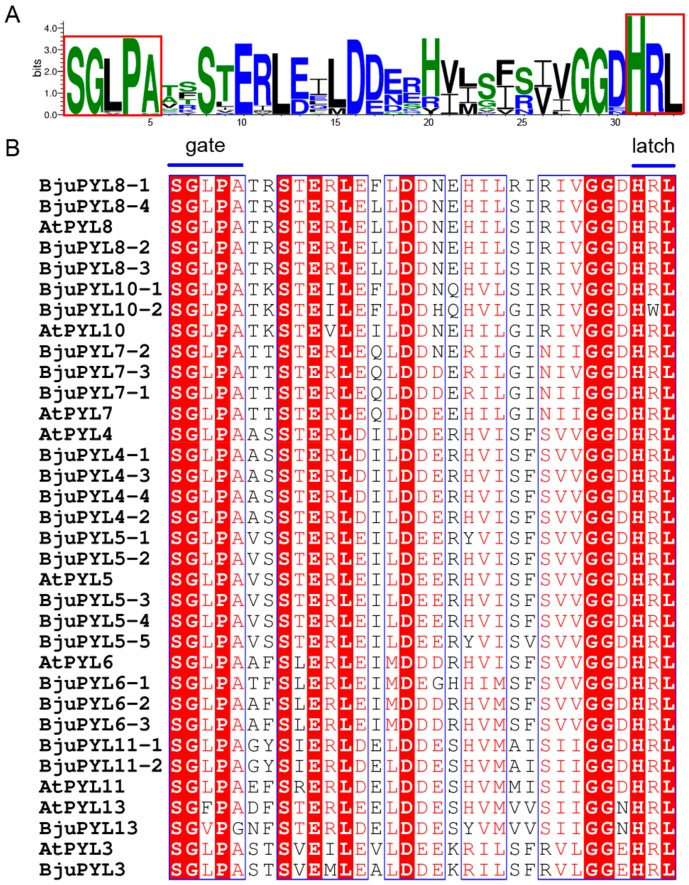
The alignment of PYL proteins and protein domain prediction. (**A**) The PYLs protein sequences were aligned by WebLogo online software, and the gate and latch residues are boxed. (**B**) The PYLs protein sequences were aligned by ESPript online software. Conserved residues are highlighted, and the gate and latch residues are noted.

The PYL protein sequences were aligned by ESPript 3.0 software (http://espript.ibcp.fr/ESPript/cgi-bin/ESPript.cgi) [20]. The results showed that the peptide sequences of these PYLs were conserved (Figure S2). The protein sequence identities among PYL3s, PYL4s, PYL5s, PYL6s, PYL7s, PYL8s, PYL10s, PYL11s, and PYL13s were more than 83.33%, 83.81%, 82.84%, 76.78%, 78.14%, 81.68%, 82.70%, 80.98%, and 79.88%, respectively (Figure S2). Moreover, the SGLPA (gate) and HRL (latch) sequences were invariant among the PYL family members, indicating that the gate and latch mechanism was likely to be a common feature of these receptors and they might be typical ABA receptors (Figure 3A,B) [21].

### 3.4. The Promoter cis-Acting Regulatory Elements Prediction of BjuPYLs

**Figure 4:**
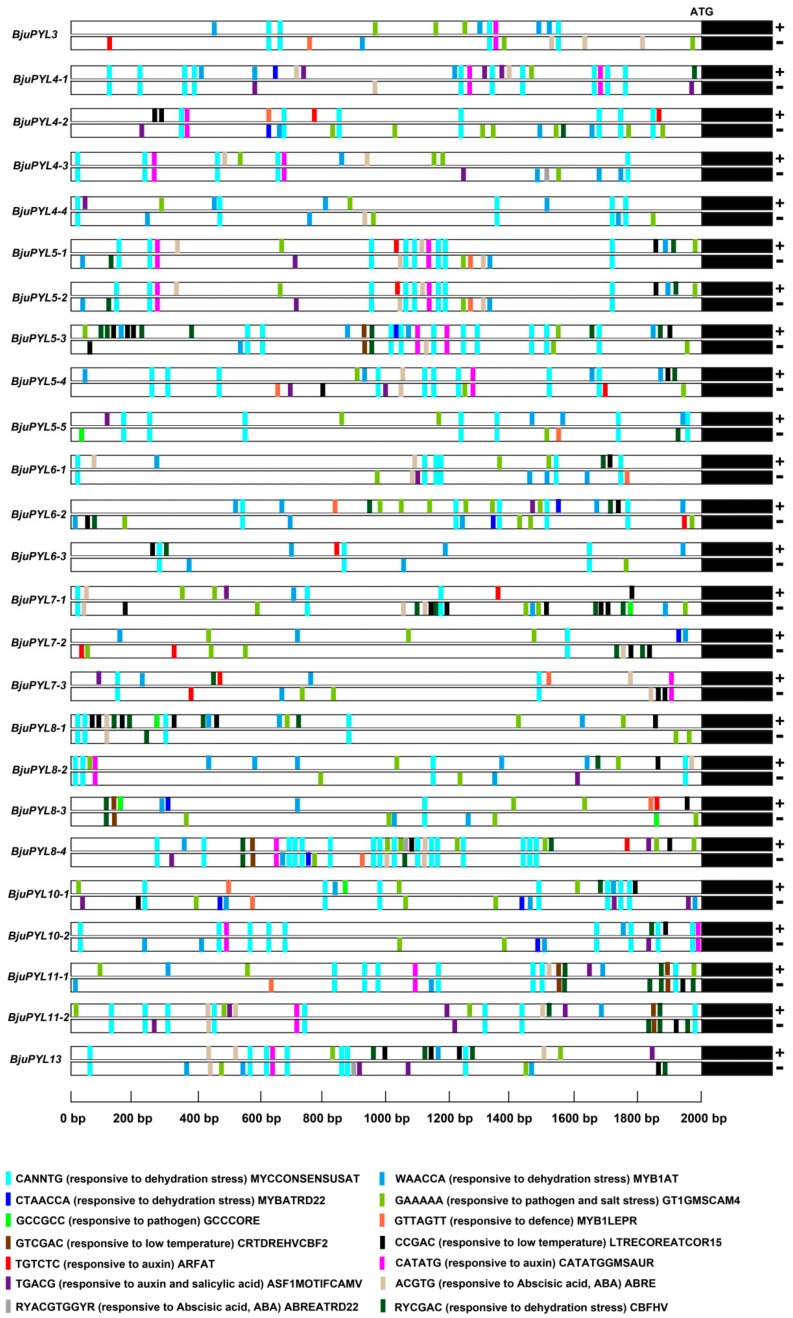
The promoter *cis*-elements analysis of *BjuPYLs*. The 2 kb DNA fragments upstream of the ATG staring code of *BjuPYLs* were analyzed using online analysis software PlantCARE (http://bioinformatics.psb.ugent.be/webtools/plantcare/html/) and PLACE (https://sogo.dna.affrc.go.jp/cgi-bin/sogo.cgi?lang=en&pj=640&action=page&page=newplace).

To further understand the potential roles of *BjuPYLs* in *B. juncea* var. *tumida* and how the genes’ expression is regulated, we chose the 2000 bp DNA fragment upstream of the ATG start code as the promoter sequences and performed the promoter *cis*-elements analysis using online software of PlantCARE and PLACE. According to the result, the promoters of *BjuPYLs* contained hormone-related elements, such as ABRE (ACGTG, responsive to Abscisic acid) [22], ARFAT (TGTCTC, responsive to auxin) [23], GMSAUR (CATATG, responsive to auxin) [24], ASF1MOTIFCAMV (TGACG, responsive to auxin and salicylic acid) [25], and ABREATRD22 (RYACGTGGYR, responsive to Abscisic acid) [26] (Figure 4). In addition, the *BjuPYLs* promoters also contained stressed-related elements, such as MYCCONSENSUSAT (CANNTG, responsive to dehydration stress) [27], MYB1AT (WAACCA, responsive to dehydration stress) [27], MYBATRD22 (CTAACCA, responsive to dehydration stress) [28], CBFHV (RYCGAC, responsive to dehydration stress) [29], GT1GMSCAM4 (GAAAAA, responsive to pathogen and salt stress) [30], GCCCORE (GCCGCC, responsive to pathogen) [31], MYB1LEPR (GTTAGTT, responsive to defence) [32], CRTDREHVCBF2 (GTCGAC, responsive to low-temperature) [33], and LTRECOREATCOR15 (CCGAC, responsive to low-temperature) [34] (Figure 4). Together, the promoters of *BjuPYLs* contained diversities of *cis*-elements responsive to ABA, auxin, SA (salicylic acid), dehydration stress, pathogen, salt stress, and low-temperature, indicating that the *BjuPYLs* genes might be involved in the regulation of the response of *B. juncea* var. *tumida* to hormone and stresses.

### 3.5. The Tissue-Specific Expression Pattern Analysis of BjuPYLs

**Figure 5:**
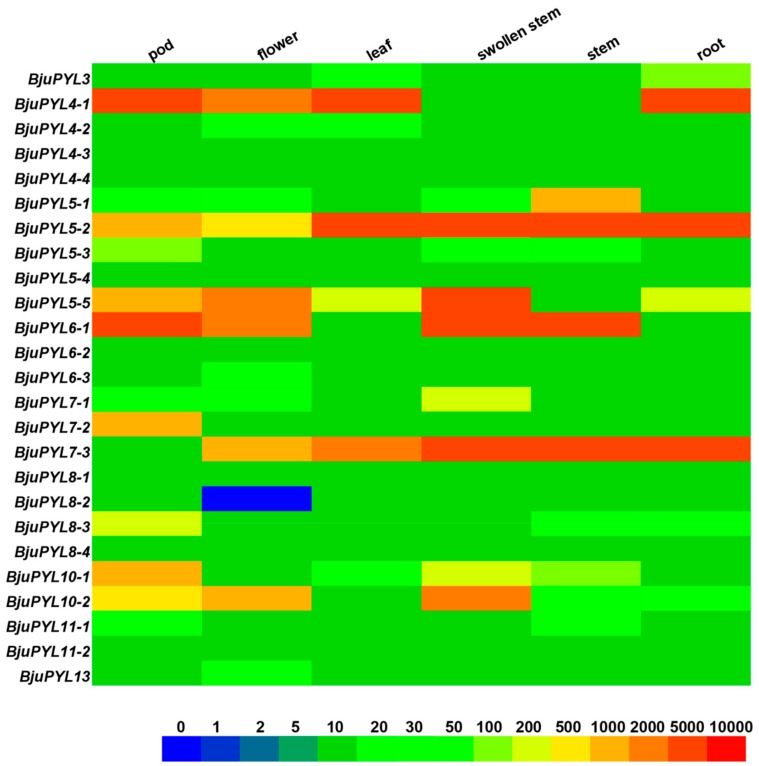
Expression levels of *BjuPYLs* in different tissues. Tissue-specific expression pattern of *BjuPYLs* was analyzed by qPCR. *BjuActin3* was used as internal control. The boxes display the gene expression levels, and different colors represent different expression levels.

To investigate the tissue-specific expression patterns of *BjuPYLs*, we analyzed the genes’ expression levels at different growth stages and tissues (root, stem, swollen stem, leaf, pod, and flower) using qRT-PCR. The results showed that *BjuPYL4-1* and *BjuPYL6-1* highly expressed in pod; *BjuPYL4-1* and *BjuPYL5-2* highly expressed in leaf; *BjuPYL5-2*, *BjuPYL5-5*, *BjuPYL6-1*, and *BjuPYL7-3* highly expressed in swollen stem; *BjuPYL5-2*, *BjuPYL6-1*, and *BjuPYL7-3* highly expressed in stem; *BjuPYL4-1*, *BjuPYL5-2*, and *BjuPYL7-3* highly expressed in root, indicating that different *BjuPYLs* might be involved in different growth and development stages, and the expression patterns of *BjuPYLs* were existence of space-time specificity (Figure 5). In contrast, the expression levels of *BjuPYL4-3*, *BjuPYL4-4*, *BjuPYL5-4*, *BjuPYL6-2*, *BjuPYL6-3*, *BjuPYL8-1*, *BjuPYL8-2*, *BjuPYL8-4*, and *BjuPYL11-2* were very low, with nearly no expression in all the tissues, indicating that these *BjuPYLs* genes had limited function during plant growth and development (Figure 5). Interestingly, we found that the expression level of *BjuPYL5-5* was much higher in the swollen stem than that in the stem, suggesting that *BjuPYL5-5* might play a role in regulating stem swelling (Figure 5).

### 3.6. The Gene Expression Levels of BjuPYLs in *B. juncea* var. *tumida* Under Abiotic Stress

**Figure 6:**
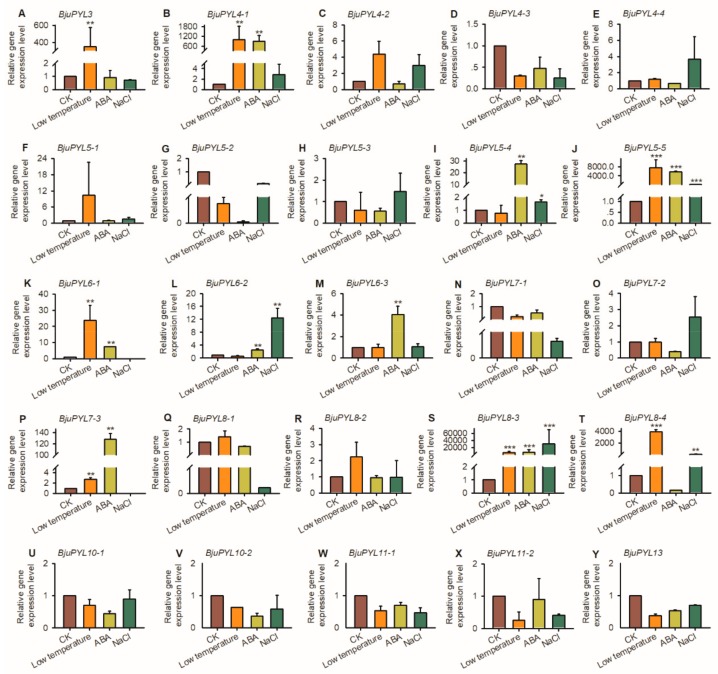
The expression patterns of *BjuPYLs* under abiotic stresses. Total RNA was extracted from 7-day-old seedlings treated with 200 mM NaCl, 50 μM ABA, and at low-temperature (4 °C) for 3 h. Data were normalized to the expression level of *BjuActin3*. The values are means ± standard error. Three independent biological repeats were performed. CK: Control Check; ABA: Abscisic acid.

To further explore the expression levels of *BjuPYLs* in *B. juncea* var. *tumida* under abiotic stresses treatment, qRT-PCR was performed using the 7-day-old seedlings treated with 200 mM NaCl, 50 μM ABA, and at low-temperature (4 °C) for 3 h. Under low-temperature stress condition, the transcript levels of *BjuPYL3*, *BjuPYL4-1*, *BjuPYL5-5*, *BjuPYL6-1*, *BjuPYL7-3*, *BjuPYL8-3*, and *BjuPYL8-4* were induced significantly; however, there were no obvious expression differences between CK (control check) and low-temperature treatment of other *BjuPYLs*, indicating that *BjuPYL3*, *BjuPYL4-1*, *BjuPYL5-5*, *BjuPYL6-1*, *BjuPYL7-3*, *BjuPYL8-3*, and *BjuPYL8-4* regulated the response of *B. juncea* var. *tumida* to low-temperature stress (Figure 6). *BjuPYL4-1*, *BjuPYL5-4*, *BjuPYL5-5*, *BjuPYL6-1*, *BjuPYL6-2*, *BjuPYL6-3*, *BjuPYL7-3*, and *BjuPYL8-3* were induced significantly after ABA treatment; in contrast, the gene expression levels of other *BjuPYLs* were stable, suggesting *BjuPYL4-1*, *BjuPYL5-4*, *BjuPYL5-5*, *BjuPYL6-1*, *BjuPYL6-2*, *BjuPYL6-3*, *BjuPYL7-3*, and *BjuPYL8-3* might be involved in ABA signaling pathway (Figure 6). Under NaCl treatment, *BjuPYL5-4*, *BjuPYL5-5*, *BjuPYL6-2*, *BjuPYL8-3*, and *BjuPYL8-4* were highly induced by salt stress, indicating that these *BjuPYLs* play roles in plant response to salt stress (Figure 6). Taken together, the expression patterns of *BjuPYLs* changed under NaCl, ABA, and low-temperature treatments, indicating that *BjuPYLs* in *B. juncea* var. *tumida* might be important candidates for regulating plant tolerance to abiotic stresses.

### 3.7. The Gene Expression Levels of BjuPYLs in *B. juncea* var. *tumida* under Pathogen Treatment

**Figure 7:**
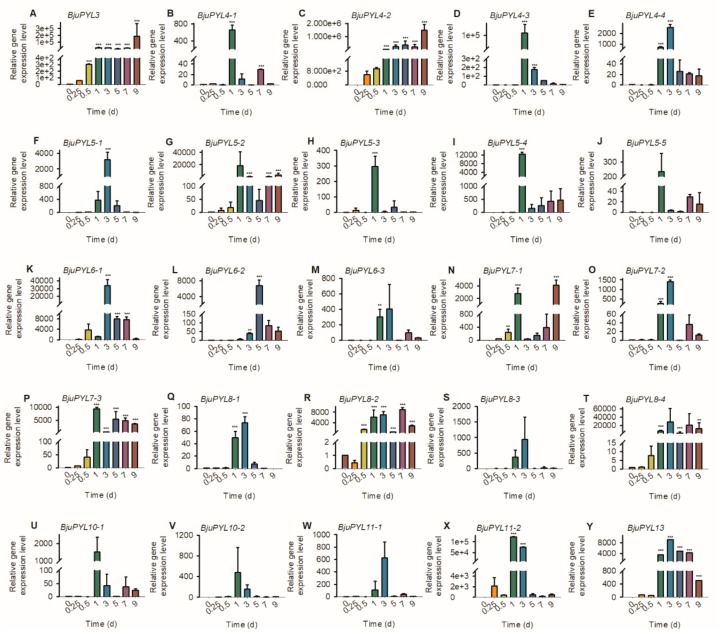
The expression patterns of *BjuPYLs* under pathogen treatment. Total RNA was extracted from 2-week-old seedlings treated with *P. brassicae* for the indicated time points. Data were normalized to the expression level of *BjuActin3*. The values are means ± standard error. Three independent biological repeats were performed.

*P. brassicae* is a main and serious pathogen of *B. juncea* var. *tumida*, which usually results in the formation of clubroot and restricts the growth and development of crucifer plants. To investigate the function of *BjuPYLs* during plant response to *P. brassicae*, 2-week-old seedlings were treated with *P. brassicae* (OD_600_ = 0.07) for 0 d, 0.25 d, 0.5 d, 1 d, 3 d, 5 d, 7 d, and 9 d. qRT-PCR assay was performed, and the result showed that gene expression levels of *BjuPYL3*, *BjuPYL4s*, *BjuPYL5s*, *BjuPYL6s*, *BjuPYL7s*, *BjuPYL8s*, and *BjuPYL13* were highly induced by *P. brassicae*, especially at 1 d and 3 d after pathogen treatment; in contrast, the other *BjuPYLs* showed similar expression levels after *P. brassicae* treatment (Figure 7). Taken together, *BjuPYLs* induced by pathogen (*P. brassicae*) treatment might be important candidates for regulating plant response to *P. brassicae*.

### 3.8. The Expression Patterns of BjuPYLs in *B. juncea* var. *tumida* During Stem Swelling Stages

**Figure 8:**
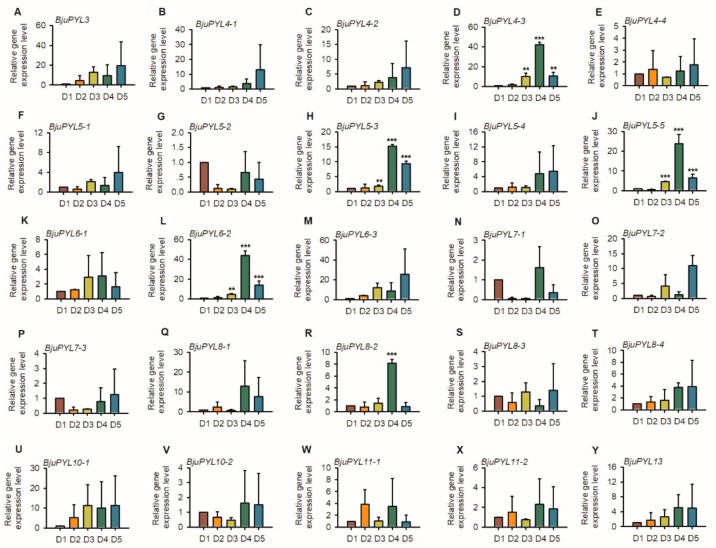
The expression patterns of *BjuPYLs* at different stages of stem swelling. Total RNA was extracted from the seedlings of D1, D2, D3, D4, and D5 stages. Data were normalized to the expression level of *BjuActin3*. The values are means ± standard error. Three independent biological repeats were performed.

To further explore the roles of *BjuPYLs* in regulating the stem swelling of *B. juncea* var. *tumida*, the qRT-PCR assay was performed. We collected the samples of *B. juncea* var. *tumida*, which were grown in the field at different growth stages, and named the samples as D1 (the stems of 1-month-old seedlings, six leaf stage), D2 (the stems of 2-month-old seedlings, primary stage of stem swelling), D3 (the stems of 3-month-old seedlings, early stage of stem swelling), D4 (the stems of 4.5-month-old seedlings, fast-growing stage of stem swelling), and D5 (the stems of 5-month-old seedlings, last stage of stem swelling). The qPCR result showed that *BjuPYL4-3*, *BjuPYL5-3*, *BjuPYL5-5*, *BjuPYL6-2*, and *BjuPYL8-2* were induced with the stem swelling, and at D4 stage (fast-growing stage of stem swelling), the expression levels of these *BjuPYL* genes were highest, and at D5 stage (last stage of stem swelling), the expression levels decreased, indicating that *BjuPYL4-3*, *BjuPYL5-3*, *BjuPYL5-5*, *BjuPYL6-2*, and *BjuPYL8-2* might be involved in regulating stem swelling of *B. juncea* var. *tumida* (Figure 8).

## 4. Discussion

The ABA receptors PYR/PYL/RCAR are core regulatory components of the ABA signaling pathway, which functions for ABA perception and signaling [6,12,16,35]. In our study, we identified 25 ABA receptor family genes in the *B. juncea* var. *tumida* genome and analyzed the functions of *BjuPYLs* in the regulation of *B. juncea* var. *tumida* responding to abiotic stresses, biotic stresses, and stem swelling. We noticed that 25 *PYL* homologous genes to 14 *Arabidopsis PYL* genes were found in *B. juncea* var. *tumida*, and the phylogenetic analysis using the PYL protein sequences was consistent with that of the *PYL* genomic sequences, indicating that the *PYL* family genes were conserved in *Arabidopsis* and *B. juncea* var. *tumida* (Figure 2, Figure S1). Most of the *PYLs* had more than two homologs; however, *PYR1*/*PYL1*/*PYL2*/*PYL9*/*PYL12* did not have homologous genes, and *PYL3*/*PYL13* only had one homologous gene in *B. juncea* var. *tumida* genome. The loss or not duplication of homologs suggests that these homologous genes may perform a functional redundancy or divarication during the evolutionary process. The expansion and loss of some PYL family genes in the *B. juncea* var. *tumida* genome suggest their possible functional differentiation in response to environmental conditions. The number of PYL genes was possibly sufficient for *B. juncea* var. *tumida* against stress from the outer environment during the long evolutionary process [36]. The losses of genes during the genome duplication event also frequently exist in other species, such as the *PYLs* in rice, *PYLs* in *Gossypium*, and the chitinase family genes in *B. rapa* [36,37,38].

*B. juncea* var. *tumida* is an allotetraploid species resulted from hybridization between *B. rapa* and *B. nigra* following with genome duplication [17]. In *Arabidopsis thaliana*, 14 *PYLs* genes were identified [6,10,11,12]. According to our results, 25 *BjuPYLs* were found in the genome of *B. juncea* var. *tumida*. *PYL4*, *PYL5*, and *PYL8* had four homologous genes located in A sub-genome and B sub-genome; *PYL6* and *PYL7* had three homologous genes located in A sub-genome and B sub-genome; *PYL10* and *PYL11* had two homologous genes located in A sub-genome and B sub-genome; *PYL3* and *PYL13* only had one homologous gene located in B sub-genome B05 and A sub-genome A01, respectively (Figure 1, Table 1). The comparable homologous gene numbers in A sub-genome and B sub-genome indicated that the *B. juncea* var. *tumida* genome experienced co-linearity gene duplication [36].

The roles of PYLs in regulating plant response to abiotic stresses have been investigated in many plants. In rice, a total of 13 *OsPYLs* were identified, and expressions of most *OsPYLs* were detected in all tissues. *OsPYL2* and *OsPYL9* expressed highly in stem, leaf, and embryo; *OsPYL3* mainly expressed in stem and leaf; *OsPYL5* had a higher expression level in leaf; *OsPYL7*, *OsPYL8* had a higher expression level in embryo. The gene expression levels of *OsPYLs* under ABA treatment were regulated differently, with the downregulation of *OsPYL1*, *OsPYL2/9*, and *OsPYL3*; the upregulation of *OsPYL4*; the stable expression of *OsPYL5*, *OsPYL7/8*, and *OsPYL10* [37]. In *Gossypium*, 21, 20, 40, and 39 *PYL* genes were identified in the genomes of *Gossypium arboretum*, *Gossypium raimondii*, *Gossypium hirsutum*, and *Gossypium barbadense*, respectively. The transcription levels of many *GhPYLs* were inhibited by ABA treatment and induced by osmotic stress [38]. In this study, according to the results of promoter *cis*-elements analysis, all the *BjuPYLs* promoters contained diversities of *cis*-elements responsive to plant hormones (ABA, auxin, and SA), abiotic stresses (drought, cold, and salt stresses), and pathogen stresses, indicating that the *BjuPYLs* were regulated by hormone, abiotic stresses, and biotic stresses (Figure 4). The gene expression levels of *BjuPYL4-3*, *BjuPYL4-4*, *BjuPYL5-4*, *BjuPYL6-2*, *BjuPYL6-3*, *BjuPYL8-1*, *BjuPYL8-2*, *BjuPYL8-4*, and *BjuPYL11-2* were very low in all tissues, indicating that these *BjuPYLs* genes had limited function in the regulation of plant growth and development (Figure 5). According to the results, we analyzed the expression levels of *BjuPYLs* using qPCR assay under various stresses. The results showed that *BjuPYLs* were induced by NaCl, low-temperature, and ABA, especially for *BjuPYL5-5* and *BjuPYL8-3*, suggesting that *BjuPYLs* played roles in plant response to abiotic stress (Figure 6). *P. brassicae* is a crucial pathogen, which leads to the formation of clubroot. The induction of *BjuPYL3*, *BjuPYL4-2*, *BjuPYL5-2*, *BjuPYL6-1*, *BjuPYL7-3*, *BjuPYL8s*, and *BjuPYL13* by *P. brassicae* indicated that they might be involved in plant response to *P. brassicae* (Figure 7). Interestingly, we also found that *BjuPYL4-3*, *BjuPYL5-3*, *BjuPYL5-5*, *BjuPYL6-2*, and *BjuPYL8-2* were highly induced at D4 stage (the fast-growing stage), suggesting that these *BjuPYLs* might play roles in stem swelling of *B. juncea* var. *tumida* (Figure 8). In conclusion, our study identified 25 *BjuPYLs* in *B. juncea* var. *tumida* genome and analyzed their transcript levels under biotic stress, abiotic stresses, and different development stages, indicating that the *BjuPYLs* might potentially be utilized for improving the tolerance of *B. juncea* var. *tumida* to stresses and regulating stem swelling.

## 5. Conclusions

In this study, a total of 25 *PYL* homologous genes were identified in the *B. juncea* var. *tumida* genome. Based on the bioinformatics analysis, *PYL* homologous genes shared similar gene characteristics and high conservation. We also found that all the promoters of *BjuPYLs* contained hormone and stress-related *cis*-elements. Gene expression analysis showed that the *BjuPYLs* were induced by abiotic stress (NaCl, low-temperature, and ABA) and biotic stress (*P. brassicae*), and these ABA receptors also played roles in regulation stem swelling in *B. juncea* var. *tumida*. Our results indicated that *BjuPYLs* played a crucial role in plant response to stresses and organ development, and the study laid a foundation for further investigations of PYL family genes in *B. juncea* var. *tumida*.

## Figures and Tables

**Table 1:** The *BjuPYLs* family members in *B. juncea* var. *tumida.*

Group	Gene Name	Locus	Sequence ID	Exon	Start (bp)	End (bp)	Genomics (bp)	CDS (bp)	Protein (aa)	pl	MW (kD)
*AtPYL3*	*BjuPYL3*	B05	*BjuB025977*	1	59063131	59062526	606	606	202	9.12	22.52
*AtPYL4*	*BjuPYL4-1*	B06	*BjuB020198*	1	4327438	4326815	624	624	208	7.08	22.48
	*BjuPYL4-2*	B08	*BjuB016454*	1	21070827	21071450	624	624	208	6.43	22.49
	*BjuPYL4-3*	A04	*BjuA000105*	1	22667580	22668194	615	615	205	6.22	21.99
	*BjuPYL4-4*	B01	*BjuB026911*	1	5965187	5964576	612	612	204	6.22	21.98
*AtPYL5*	*BjuPYL5-1*	A10	*BjuA039937*	1	18355046	18354435	612	612	204	6.08	22.75
	*BjuPYL5-2*	B02	*BjuB048564*	1	51651202	51650591	612	612	204	5.82	22.72
	*BjuPYL5-3*	A03	*BjuA009007*	1	1129007	1129615	609	609	203	5.80	22.71
	*BjuPYL5-4*	B05	*BjuB040841*	1	1261985	1262593	609	609	203	6.13	22.64
	*BjuPYL5-5*	A02	*BjuA040927*	1	1002880	1003470	591	591	197	6.03	22.04
*AtPYL6*	*BjuPYL6-1*	A03	*BjuA010539*	1	12296114	12295479	636	636	212	6.56	23.49
	*BjuPYL6-2*	B01	*BjuB042092*	1	4270506	4271120	615	615	205	6.09	22.76
	*BjuPYL6-3*	B01	*BjuB042125*	2	4517378	4518303	926	663	221	6.70	24.25
*AtPYL7*	*BjuPYL7-1*	B08	*BjuB046026*	3	23036589	23035860	730	570	190	7.12	21.46
	*BjuPYL7-2*	A03	*BjuA011393*	3	17694212	17694945	734	582	194	6.30	21.73
	*BjuPYL7-3*	B08	*BjuB017238*	3	41743391	41744197	807	582	193	6.13	21.73
*AtPYL8*	*BjuPYL8-1*	Contig	*BjuO010274*	3	98737	99690	954	552	184	6.07	20.89
	*BjuPYL8-2*	B08	*BjuB041138*	3	15536917	15535680	1238	558	186	6.30	20.89
	*BjuPYL8-3*	A02	*BjuA006960*	3	8573368	8572564	805	567	189	6.24	21.29
	*BjuPYL8-4*	A10	*BjuA015299*	3	7690566	7691613	1048	555	185	6.07	21.03
*AtPYL10*	*BjuPYL10-1*	A01	*BjuA004705*	3	11241174	11241943	770	552	184	5.61	20.65
	*BjuPYL10-2*	B05	*BjuB040453*	3	6546487	6545721	767	552	184	6.25	21.06
*AtPYL11*	*BjuPYL11-1*	B02	*BjuB036696*	1	54967275	54966790	486	486	162	5.41	18.05
	*BjuPYL11-2*	A06	*BjuA024968*	1	29659710	29660219	510	510	170	5.21	18.75
*AtPYL13*	*BjuPYL13*	A01	*BjuA003983*	1	5691022	5691519	498	498	166	5.01	18.38

pI: Isoelectric point; MW: molecular weight; CDS: coding sequence.

## Supplementary Materials

The following are available online at https://www.mdpi.com/2073-4425/10/6/470/s1; Table S1: The primers used in this study, Figure S1: The phylogenic tree of *BjuPYLs* and *AtPYLs*, Figure S2: The alignment of PYLs proteins and protein domain prediction.

## References

[1] Finkelstein R.R., Gampala S.S., Rock C.D. (2002). Abscisic acid signaling in seeds and seedlings. Plant Cell.

[2] Koornneef M., Hanhart C.J., Hilhorst H.W., Karssen C.M. (1989). In vivo inhibition of seed development and reserve protein accumulation in recombinants of abscisic acid biosynthesis and responsiveness mutants in *Arabidopsis thaliana*. Plant Physiol..

[3] Raghavendra A.S., Gonugunta V.K., Christmann A., Grill E. (2010). ABA perception and signalling. Trends Plant Sci..

[4] Zhu J.K. (2002). Salt and drought stress signal transduction in plants. Annu. Rev. Plant Biol..

[5] Ton J., Flors V., Mauch-Mani B. (2009). The multifaceted role of ABA in disease resistance. Trends Plant Sci..

[6] Cutler S.R., Rodriguez P.L., Finkelstein R.R., Abrams S.R. (2010). Abscisic acid: Emergence of a core signaling network. Annu. Rev. Plant Biol..

[7] Kim T.H., Bohmer M., Hu H., Nishimura N., Schroeder J.I. (2010). Guard cell signal transduction network: Advances in understanding abscisic acid, CO_2_, and Ca^2+^ signaling. Annu. Rev. Plant Biol..

[8] Kong L., Cheng J., Zhu Y., Ding Y., Meng J., Chen Z., Xie Q., Guo Y., Li J., Yang S. (2015). Degradation of the ABA co-receptor ABI1 by PUB12/13 U-box E3 ligases. Nat. Commun..

[9] Fujii H., Chinnusamy V., Rodrigues A., Rubio S., Antoni R., Park S.Y., Cutler S.R., Sheen J., Rodriguez P.L., Zhu J.K. (2009). In vitro reconstitution of an abscisic acid signalling pathway. Nature.

[10] Ma Y., Szostkiewicz I., Korte A., Moes D., Yang Y., Christmann A., Grill E. (2009). Regulators of PP2C phosphatase activity function as abscisic acid sensors. Science.

[11] Park S.Y., Fung P., Nishimura N., Jensen D.R., Fujii H., Zhao Y., Lumba S., Santiago J., Rodrigues A., Chow T.F. (2009). Abscisic acid inhibits type 2C protein phosphatases via the PYR/PYL family of START proteins. Science.

[12] Gonzalez-Guzman M., Pizzio G.A., Antoni R., Vera-Sirera F., Merilo E., Bassel G.W., Fernandez M.A., Holdsworth M.J., Perez-Amador M.A., Kollist H. (2012). *Arabidopsis* PYR/PYL/RCAR receptors play a major role in quantitative regulation of stomatal aperture and transcriptional response to abscisic acid. Plant Cell.

[13] Lackman P., Gonzalez-Guzman M., Tilleman S., Carqueijeiro I., Pérez A.C., Moses T., Seo M., Kanno Y., Häkkinen S.T., Van-Montagu M.C. (2011). Jasmonate signaling involves the abscisic acid receptor PYL4 to regulate metabolic reprogramming in *Arabidopsis* and tobacco. Proc. Natl. Acad. Sci. USA.

[14] Bueso E., Rodriguez L., Lorenzo-Orts L., Gonzalez-Guzman M., Sayas E., Muñoz-Bertomeu J., Ibañez C., Serrano R., Rodriguez P.L. (2015). The single-subunit RING-type E3 ubiquitin ligase RSL1 targets PYL4 and PYR1 ABA receptors in plasma membrane to modulate abscisic acid signaling. Plant J..

[15] Kim H., Lee K., Hwang H., Bhatnagar N., Kim D.Y., Yoon I.S., Byun M.O., Kim S.T., Jung K.H., Kim B.G. (2014). Overexpression of PYL5 in rice enhances drought tolerance, inhibits growth, and modulates gene expression. J. Exp. Bot..

[16] Di F., Jian H., Wang T., Chen X., Ding Y., Du H., Lu K., Li J., Liu L. (2018). Genome-wide analysis of the PYL gene family and identification of PYL genes that respond to abiotic stress in *Brassica napus*. Genes.

[17] Yang J., Liu D., Wang X., Ji C., Cheng F., Liu B., Hu Z., Chen S., Pental D., Ju Y. (2016). The genome sequence of allopolyploid *Brassica juncea* and analysis of differential *homoeolog* gene expression influencing selection. Nat. Genet..

[18] Shi H., Wang L.L., Sun L.T., Dong L.L., Liu B., Chen L.P. (2012). Cell division and endoreduplication play important roles in stem swelling of tuber mustard (*Brassica juncea* Coss. var. *tumida* Tsen et Lee). Plant Biol..

[19] Tamura K., Peterson D., Peterson N., Stecher G., Nei M., Kumar S. (2011). MEGA5: Molecular evolutionary genetics analysis using maximum likelihood, evolutionary distance, and maximum parsimony methods. Mol. Biol. Evol..

[20] Robert X., Gouet P. (2014). Deciphering key features in protein structures with the new ENDscript server. Nucleic Acids Res..

[21] Melcher K., Ng L.M., Zhou X.E., Soon F.F., Xu Y., Suino-Powell K.M., Park S.Y., Weiner J.J., Fujii H., Chinnusamy V. (2009). A gate–latch–lock mechanism for hormone signalling by abscisic acid receptors. Nature.

[22] Nakashima K., Fujita Y., Katsura K., Maruyama K., Narusaka Y., Seki M., Shinozaki K., Yamaguchi-Shinozaki K. (2006). Transcriptional regulation of ABI3- and ABA-responsive genes including RD29B and RD29A in seeds, germinating embryos, and seedlings of *Arabidopsis*. Plant Mol. Biol..

[23] Nag R., Maity M.K., Dasgupta M. (2005). Dual DNA binding property of ABA insensitive 3 like factors targeted to promoters responsive to ABA and auxin. Plant Mol. Biol..

[24] Xu N., Hagen G., Guilfoyle T. (1997). Multiple auxin response modules in the soybean SAUR 15A promoter. Plant Sci..

[25] Redman J., Whitcraft J., Johnson C., Arias J. (2002). Abiotic and biotic stress differentially stimulate as-1 element activity in *Arabidopsis*. Plant Cell Rep..

[26] Iwasaki T., Yamaguchi-Shinozaki K., Shinozaki K. (1995). Identification of a cis -regulatory region of a gene in *Arabidopsis thaliana* whose induction by dehydration is mediated by abscisic acid and requires protein synthesis. Mol. Gen. Genet..

[27] Abe H., Urao T., Ito T., Seki M., Shinozaki K., Yamaguchi-Shinozaki K. (2003). *Arabidopsis* AtMYC2 (bHLH) and AtMYB2 (MYB) function as transcriptional activators in abscisic acid signaling. Plant Cell.

[28] Abe H., Yamaguchi-Shinozaki K., Urao T., Iwasaki T., Hosokawa D., Shinozaki K. (1997). Role of *Arabidopsis* MYC and MYB homologs in drought- and abscisic acid-regulated gene expression. Plant Cell.

[29] Xue G.P. (2002). Characterisation of the DNA-binding profile of barley HvCBF1 using an enzymatic method for rapid, quantitative and high-throughput analysis of the DNA-binding activity. Nucleic Acids Res..

[30] Park H.C., Kim M.L., Kang Y.H., Jeon J.M., Yoo J.H., Kim M.C., Park C.Y., Jeong J.C., Moon B.C., Lee J.H. (2004). Pathogen- and NaCl-induced expression of the SCaM-4 promoter is mediated in part by a GT-1 box that interacts with a GT-1-like transcription factor. Plant Physiol..

[31] Brown R.L., Kazan K., McGrath K.C., Maclean D.J., Manners J.M. (2003). A role for the GCC-box in jasmonate-mediated activation of the PDF1.2 gene of *Arabidopsis*. Plant Physiol..

[32] Chakravarthy S. (2003). The tomato transcription factor Pti4 regulates defense-related gene expression via GCC box and non-GCC box cis elements. Plant Cell.

[33] Xue G.P. (2003). The DNA-binding activity of an AP2 transcriptional activator HvCBF2 involved in regulation of low-temperature responsive genes in barley is modulated by temperature. Plant J..

[34] Baker S.S., Wilhelm K.S., Thomashow M.F. (1994). The 5′-region of *Arabidopsis thaliana* cor15a has cis-acting elements that confer cold-, drought- and ABA-regulated gene expression. Plant Mol. Biol..

[35] Cheng C., Wang Z., Ren Z., Zhi L., Yao B., Su C., Liu L., Li X. (2017). SCFAtPP2-B11 modulates ABA signaling by facilitating SnRK2.3 degradation in *Arabidopsis thaliana*. PLoS Genet..

[36] Chen J., Piao Y., Liu Y., Li X., Piao Z. (2018). Genome-wide identification and expression analysis of chitinase gene family in *Brassica rapa* reveals its role in clubroot resistance. Plant Sci..

[37] Tian X., Wang Z., Li X., Lv T., Liu H., Wang L., Niu H., Bu Q. (2015). Characterization and functional analysis of pyrabactin resistance-like abscisic acid receptor family in rice. Rice.

[38] Zhang G., Lu T., Miao W., Sun L., Tian M., Wang J., Hao F. (2017). Genome-wide identification of ABA receptor PYL family and expression analysis of *PYLs* in response to ABA and osmotic stress in *Gossypium*. PeerJ.

