# Supplementary material for: Genome-Wide Identification and Gene Expression Analysis of ABA Receptor Family Genes in Brassica juncea var. tumida
Source: Genes (Basel). 2019 Jun 20;10(6):470. doi: 10.3390/genes10060470 (PMC6628100; doi:10.3390/genes10060470)
Supplement: Supplementary file 1 [file genes-10-00470-s001.pdf]

Table S1. The primers used in this study

Primer Name	Sequence
qRT-BjuPYL3-F	CTACCTTCTAGCCACGCCC
qRT-BjuPYL3-R	GCGTTCTGATGATAGGATC
qRT-BjuPYL4-1-F	GTCTACGTCAAGTCCACGTC
qRT-BjuPYL4-1-R	GTCCCGGAGACTGGAGAAGAG
qRT-BjuPYL4-2-F	CCGCAGCTCGTTTCCATGCC
qRT-BjuPYL4-2-R	CGTCGTCGAGGATGTCTAAAC
qRT-BjuPYL4-3-F	CCGCCGTGATTCAAGAAATC
qRT-BjuPYL4-3-R	ATAGTTAGAGAGCCGGTGATC
qRT-BjuPYL4-4-F	CACTCTTCTCCGGTGTCC
qRT-BjuPYL4-4-R	CCCCTCCGCCGCAGATAAC
qRT-BjuPYL5-1-F	GATCCACGCGCCGCCCGGG
qRT-BjuPYL5-1-R	GCTTATCACGTAACGCTCTTC
qRT-BjuPYL5-2-F	ACACGCGCCGCCCGAGTCTG
qRT-BjuPYL5-2-R	GCTTATCACGTGCCTCTCC
qRT-BjuPYL5-3-F	ACATGGCTCCGACGCCCCC
qRT-BjuPYL5-3-R	GGGACCAGACGGACTCGGGTG
qRT-BjuPYL5-4-F	GCCCTCCTGCGTCGTTTCGAC
qRT-BjuPYL5-4-R	CCCACGACGCTGAAGCTG
qRT-BjuPYL5-5-F	ATGACATCACCGGTGCAGCTCC
qRT-BjuPYL5-5-R	CCGAGGAGCAGCACTTGCCTG
qRT-BjuPYL6-1-F	CGCTCCGTCTTCAGCCGTG
qRT-BjuPYL6-1-R	ACTCATGATGTGGTGACCC
qRT-BjuPYL6-2-F	GCCAACATCTCTACAATTT
qRT-BjuPYL6-2-R	GCACTGAGAGGGTTCCACAAC
qRT-BjuPYL6-3-F	CCGGGCCGGTCCTTCTGTG
qRT-BjuPYL6-3-R	CGACCAGACGGCGGAAGCAGG
qRT-BjuPYL7-1-F	GGAGGAGATCATCGTAGAC
qRT-BjuPYL7-1-R	GGATGACCATGAACTGTAC
qRT-BjuPYL7-2-F	GAGCTCTTGTCACGGCGCAG
qRT-BjuPYL7-2-R	CCGATCTCAGGATGACCATGG
qRT-BjuPYL7-3-F	GGAGGAGATCATCGTAGACG
qRT-BjuPYL7-3-R	GGATGACCGTGAACTGTGC
qRT-BjuPYL8-1-F	GCACGAGCTTGAGAGAGAG
qRT-BjuPYL8-1-R	CTCCATGTTTCCTTTAATC
qRT-BjuPYL8-2-F	CCACCATAAGCATGCGCTTC
qRT-BjuPYL8-2-R	CAGTTCCAATCTCCATGTTC
qRT-BjuPYL8-3-F	GAATCAGTGTAGCTCTACGG
qRT-BjuPYL8-3-R	GACTTCACATCAATTTCTCG
qRT-BjuPYL8-4-F	TAAGCATGAGCTCGAGAGG
qRT-BjuPYL8-4-R	AACCACACATCTACTAATG
qRT-BjuPYL10-1-F	GATGCACCACATGCACGGTG
qRT-BjuPYL10-1-R	CATCTACTTCTCTTACTGAAC
**Primer Name**	**Sequence**
qRT-BjuPYL10-2-F	GAGGCACCACATGCAGGAGG
qRT-BjuPYL10-2-R	AACCGTACCAATCTCAAGC
qRT-BjuPYL11-1-F	GCGATGGAAGAGAAGGATC
qRT-BjuPYL11-1-R	GGTATCAGCGAACGACATTG
qRT-BjuPYL11-2-F	GGCGGTGACCACCGTCTCC
qRT-BjuPYL11-2-R	GATTTTCGCTTGGGTGCCAT
qRT-BjuPYL13-F	GTCTCCGGCGTTCCAGGT
qRT-BjuPYL13-R	CTTGTCCGCCTCATCATCAG
qRT-BjuActin3-F	GGCTACTCTTTCACCACGAC
qRT-BjuActin3-R	GGATACCAGCATTCTCCATAC

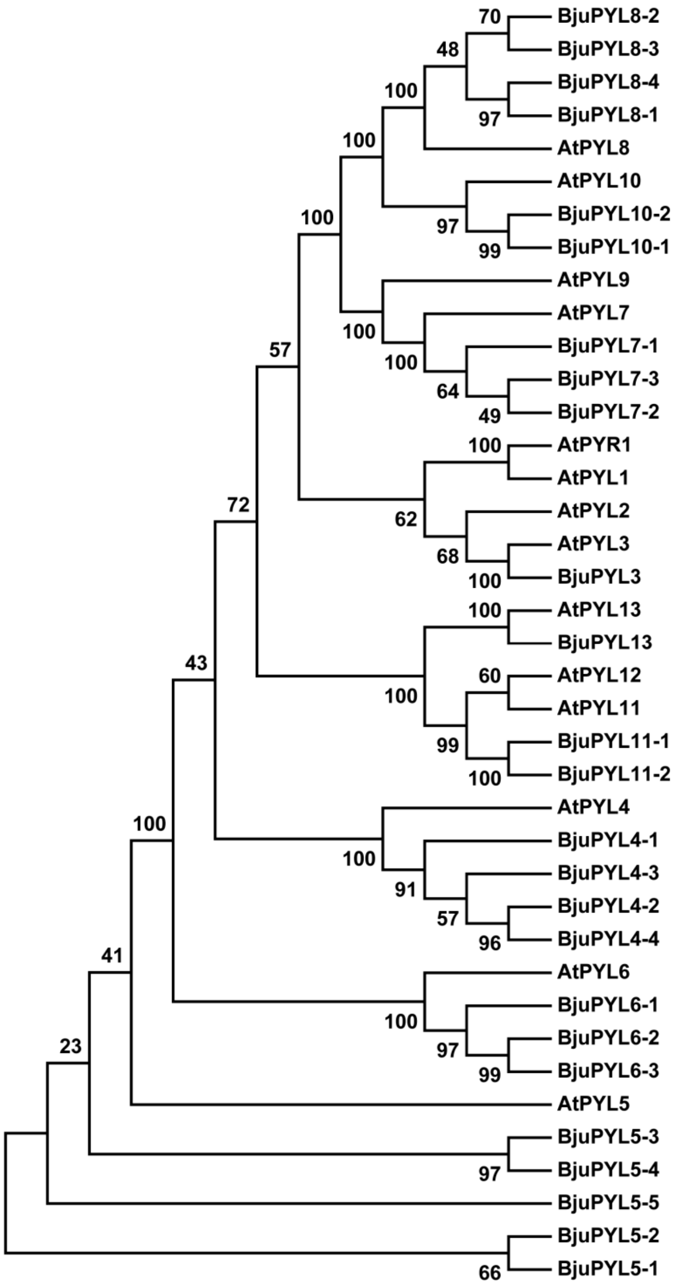

Figure S1. The phylogenic tree of *BjuPYLs* and *AtPYLs*. The phylogenic tree was built with the neighbor-joining (NJ) method using the genomic sequences.

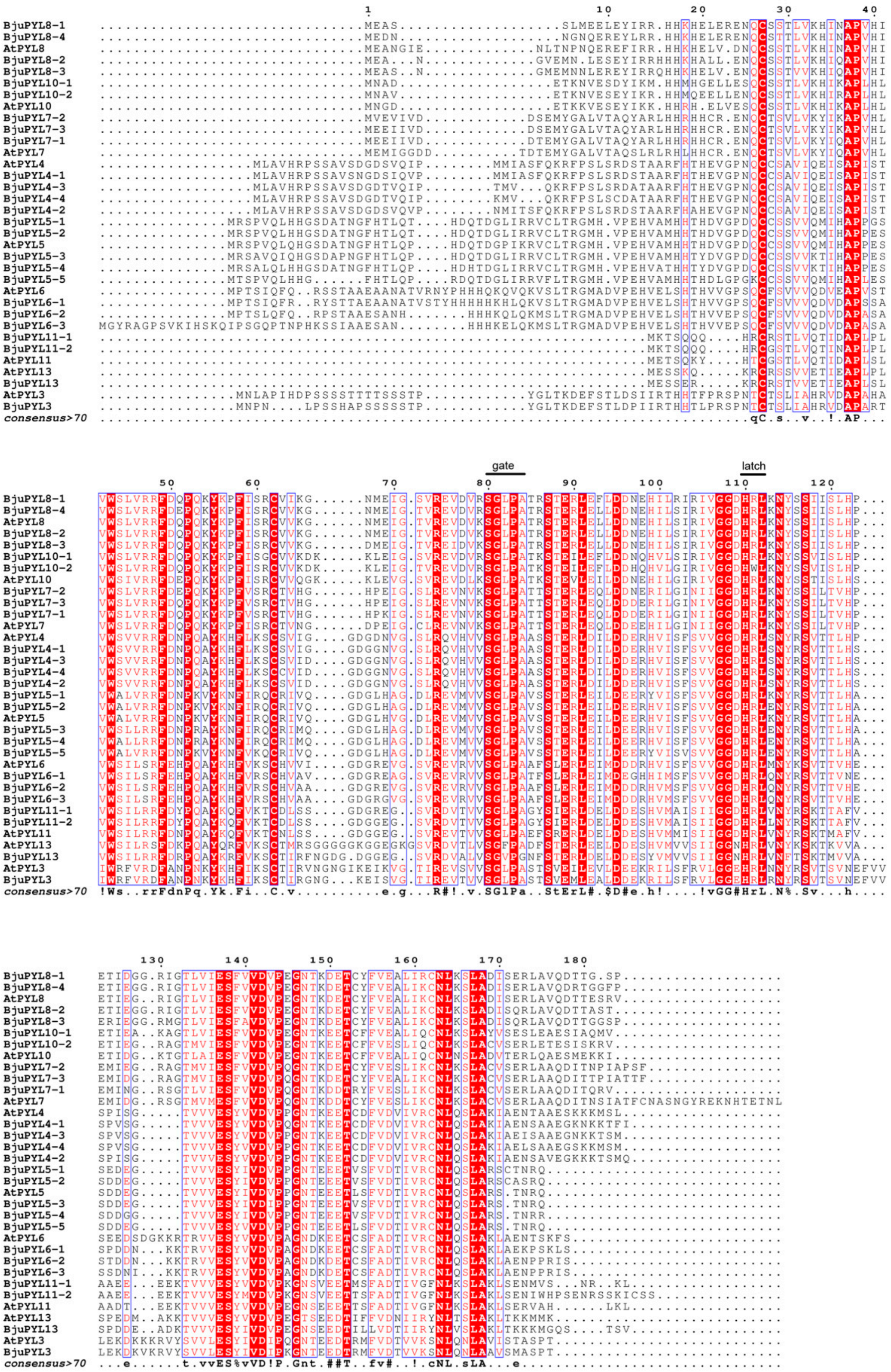

Figure S2. The alignment of PYLs proteins and protein domain prediction. The PYLs protein sequences were aligned by ESPript online software. Conserved residues are highlighted and the gate and latch residues are noted.
